# Glucose metabolism is differentially altered by choline and methionine in bovine neonatal hepatocytes

**DOI:** 10.1371/journal.pone.0217160

**Published:** 2019-05-29

**Authors:** Tawny L. Chandler, Heather M. White

**Affiliations:** Department of Dairy Science, University of Wisconsin-Madison, Madison, WI, United States of America; Universite du Quebec a Montreal, CANADA

## Abstract

Choline and methionine serve essential roles in the liver that may interact with glucose metabolism. Our objectives were to quantify glucose export, cellular glycogen, and expression of genes controlling oxidation and gluconeogenesis in primary bovine neonatal hepatocytes exposed to increasing concentrations of choline chloride (CC) and D,L-methionine (DLM) with or without fatty acids (FA). Primary hepatocytes isolated from 3 Holstein calves were maintained as monolayer cultures for 24 h before treatment with CC (61, 128, 2028, 4528 μmol/L) and DLM (16, 30, 100, 300 μmol/L) with or without a 1 mmol/L FA cocktail in a factorial design. After 24 h, media was harvested to quantify glucose, β-hydroxybutyrate (BHB), and cells harvested to quantify glycogen, DNA, and gene expression. No interactions between CC and DLM were detected. The potential two-way interaction between CC or DLM and FA was partitioned into three contrasts when *P* ≤ 0.20: linear without FA, linear with FA, difference of slope. Fatty acids did not affect glucose or cellular glycogen but increased pyruvate carboxylase (*PC*), cytosolic and mitochondrial phosphoenolpyruvate carboxykinase *(PEPCKc*, *PEPCKm*), and glucose-6-phosphatase (*G6PC*) expression. Increasing CC decreased glucose but increased cellular glycogen. Expression of *PC* and *PEPCKc* was increased by CC during FA treatment. Increasing DLM did not affect metabolites or *PC* expression, although *PEPCKc* was marginally decreased. Methionine did not affect *G6PC*, while CC had a marginal quadratic effect on *G6PC*. Oxidative and gluconeogenic enzymes appear to respond to FA in primary bovine neonatal hepatocytes. Increased *PC* and *PEPCKc* by CC during FA treatment suggest increased gluconeogenic capacity. Changes in *G6PC* may have shifted glucose-6-phosphate towards cellular glycogen; however, subsequent examination of G6PC protein is needed. Unaltered *PC* and marginally decreased *PEPCKc* suggest increased oxidative capacity with DLM, although BHB export was unaltered. The differential regulation supports unique effects of CC and DLM within bovine hepatocytes.

## Introduction

Hepatic metabolism responds to increased glucose demand at the onset of lactation by doubling gluconeogenesis [1]. In addition, proportional contributions of lactate and amino acids to glucose carbon increase [2] as propionate, the primary gluconeogenic precursor, is limited by a voluntary reduction in feed intake around the time of calving [3,4]. As a result, cows experience negative energy balance peripartum and respond by mobilizing fatty acids (**FA**) from adipose tissue. Fatty acids are taken up by the liver for complete oxidation as acetyl-CoA in the tricarboxylic acid (**TCA**) cycle, incomplete oxidation to ketone bodies, storage as triglycerides (**TG**), or export as very low-density lipoproteins (**VLDL**) [5]. Oxidative and gluconeogenic capacity, two competing pathways, are determined by carbon availability in the TCA cycle. Excessive hepatic FA uptake and imbalance of oxidative pathway capacity likely contributes to the development of hyperketonemia and fatty liver, two common metabolic disorders that challenge the performance and health of dairy cows [6].

Supplementing rumen-protected choline (**RPC**) and rumen-protected methionine (**RPM**) peripartum has increased post-partum production [7,8]. Previous studies have attributed production responses to the hepatoprotective effects elicited by the nutrients [8–10]. Choline is required for phosphatidylcholine synthesis and VLDL export [11], while methionine (**Met**) is an essential amino acid for protein synthesis and a precursor of intracellular antioxidants [12]. In addition to these essential roles, both choline and Met donate methyl groups to support synthesis of S-adenosylmethionine (**SAM**), the universal biological methyl donor [13]. Supplying choline and Met may improve hepatic function, in turn, affecting pathways of glucose metabolism. Indeed, observed increases in milk yield without sufficient increases in DMI with peripartal supplementation of RPC [7,14] or RPM [8,15] suggest the nutrients stimulate glucose supply to support lactogenesis and subsequent milk yield. This would be critical during a period when energy supply from intake does not meet milk energy output [16].

The hepatoprotective effects of choline and Met may directly or indirectly alter glucose metabolism in the liver and prevent metabolic disorders. Choline’s lipotropic action may prevent hepatic lipidosis which is credited for limiting gluconeogenesis [5,17], and Met supply may protect the liver from oxidative stress and inflammation that inhibits function [10]. Metabolism of both nutrients can biochemically contribute intermediates to the TCA cycle and potentially gluconeogenesis: choline via metabolism to glycine and serine during methyl carbon metabolism [18], and Met via conversion to cysteine and succinyl-CoA [19]. We hypothesized that choline and Met can alter gluconeogenic pathways in primary bovine neonatal hepatocytes and this may be altered by FA. Therefore, our objective was to investigate effects of these nutrients on cellular glycogen, glucose export, and the expression of key genes controlling gluconeogenesis in the absence and presence of FA. Considering the interaction between pathways of complete and incomplete oxidation and gluconeogenesis, β-hydroxybutyrate (**BHB**) export was quantified when hepatocytes were exposed to FA as an indicator of incomplete oxidation.

## Materials and methods

### Ethics statement

All animal use and handling protocols were approved by the University of Wisconsin-Madison College of Agricultural and Life Sciences Animal Care and Use Committee.

### Isolation and cell culture

Primary bovine hepatocytes were isolated from 3 Holstein bull calves < 7 d of age by collagenase perfusion of the caudate process as previously described [20]. Isolated cells were plated on 35-mm Corning Primaria culture plates (Fisher Scientific, Hampton, NH) in Dulbecco’s Modified Eagle’s Medium (**DMEM** 2902, 5.5 mmol/L glucose, 1.25 mmol/L sodium pyruvate; Sigma-Aldrich, St. Louis, MO) supplemented with 20% fetal bovine serum (**FBS**) and 1% antibiotic, antimycotic solution (A5955, Sigma-Aldrich, St. Louis, MO). Four hours after initial plating, media was refreshed with a 10% FBS, 1% antibiotic, antimycotic DMEM.

### Treatments

Monolayer cultures of cells were maintained for 24 h and were at least 80% confluent before exposure to treatments. As previously described [21], cells were randomly assigned to increasing concentrations of choline chloride (**CC**), D,L-Met (**DLM**), and a FA cocktail in a 4×4×2 factorial arrangement. To control the concentration of Met in treatment media, a Met-free media was made according to the formulation for low glucose DMEM (2902; Sigma-Aldrich, St. Louis, MO) without adding Met. The Met-free treatment media was fortified with a Basal Medium Eagle (**BME**) vitamin solution (6891; Sigma-Aldrich, St. Louis, MO) that contained 716 μmol/L CC and 227 μmol/L folic acid, and was completed to 1% bovine serum albumin (**BSA**; Merck Millipore, Billerica, MA) and 1% antibiotic, antimycotic solution. Sterile stock solutions of DLM were added to wells containing treatment media to achieve target concentrations of 16, 30, 100, and 300 μmol/L DLM. Because the fortified Met-free DMEM contained 28 μmol/L CC via the BME vitamin solution, sterile stock solutions of CC were added to achieve target concentrations of 61, 128, 2028, and 4528 μmol/L CC. Treatment concentrations were selected based on the limited information available regarding arterial concentrations of Met and choline metabolites when the nutrients are supplemented. Arterial plasma concentrations of methionine can increase up to 144 μmol/L when rumen-protected methionine is fed [22], and total plasma choline metabolites can reach 14,241 μmol/L depending on stage of lactation [23]. It is important to note that nutrient concentrations are greater in the portal vein, which would more closely represent what hepatocytes are exposed to; therefore, treatments of 16, 30, 100, 300 μmol/L DLM and 61, 128, 2028, and 4528 μmol/L CC were used.

Treatments were replicated with and without addition of a FA cocktail (1 mmol/L). Stocks of FA C14:0, C16:0, C18:0, C18:1n-9 *cis*, C18:2n-6 *cis*, and C18:3n-3 *cis* (Sigma-Aldrich, St. Louis, MO) were bound to BSA by the method previously described [24] and combined in molar ratios to create a FA cocktail that mimicked the profile of circulating FA observed for dairy cows at the time of calving [25]. The FA cocktail was comprised of 3% C14:0, 27% C16:0, 23% C18:0, 31% C18:1n-9 *cis*, 8% C18:2n-6 *cis*, and 8% C18:3n-3 *cis*. No additional BSA was added to treatment media supplemented with FA cocktail. All treatments were applied in triplicate in independent preparations of cells from 3 Holstein calves. All reagents used were of cell-culture grade or the highest purity available (Sigma-Aldrich, St. Louis, MO).

### Quantifying media glucose, cellular glycogen, and BHB

Twenty-four hours after treatment exposure, media was harvested from each well within a triplicate-treatment and stored at -80°C for later quantification of glucose by colorimetric assay (Autokit Glucose, Wako Diagnostics, Richmond, VA) and BHB by colorimetric assay using a commercial kit (CataChem Inc., Bridgeport, CT) utilizing an autoanalyzer (CataChemWell-T, Awareness Technologies, Westport, CT). Export of BHB was only quantified from cells that were exposed to FA treatment.

After media harvest, cells were rinsed with 1X PBS before harvest in 0.5 mL 1X PBS and stored at -80°C for subsequent isolation of cellular glycogen. Cellular glycogen was precipitated based on a previously established method [26] with modifications [27]. Briefly, cells were sonicated in 0.5 mL 1X PBS before a 50 μL aliquot was removed and diluted 1:10 with 1X TE buffer for the quantification of total DNA by a PicoGreen dsDNA quantification reagent (P-7589; Molecular Probes, Eugene, OR) as previously described [28]. Samples were assayed in triplicate and the concentration of DNA was read against a calf thymus DNA standard. An equal volume of 60% KOH saturated with sodium sulfate was added to the remaining cell homogenate and the sample was placed in a 90°C dry heat block for 30 min. Samples were cooled and 3 mL of 100% ethanol was added and glycogen precipitated overnight at 4°C. Glycogen was pelleted at 1,800 x *g* for 15 min and the supernatant discarded before the glycogen pellet was washed with 1 mL of cold 100% ethanol and pelleted again at 1,800 x *g* for 5 min. Isolated glycogen was then converted to glucose units by acid-heat hydrolysis [29]. The pellet was dried and dissolved in 1X PBS and moved to a cryogenic tube where an equal volume of 4 M HCl was added and the sample placed in a 95°C dry heat block for 2 h. The sample was cooled and neutralized with 10 M NaOH before glucose was quantified by colorimetric assay (Autokit Glucose, Wako Diagnostics, Richmond, VA).

Total media glucose, glycogen, and BHB were normalized to corresponding total DNA within each culture plate prior to averaging within triplicates. Data for media glucose and cellular glycogen were expressed relative to the lowest CC and DLM treatment without FA within each independent cell preparation, while BHB data were expressed relative to the lowest CC and DLM treatment with FA within each independent cell preparation.

### RNA isolation and qPCR

In a parallel incubation of identical treatments, media was aspirated, cells were rinsed with 1X PBS and harvested in 0.5 mL Trizol reagent (Invitrogen, Carlsbad, CA) and stored at -80°C until subsequent RNA extraction and characterization. Total RNA was isolated from each plate using Trizol reagent and sample concentration was quantified and characterized using a spectrophotometer (Synergy H1, BioTek Instruments, Inc., Winooski, VT). A 100 μL pool containing balanced quantities of RNA from each triplicate was further purified using an on-column DNase I treatment and the RNeasy Mini Kit (Qiagen Inc., Thousand Oaks, CA). Cleaned RNA was re-quantified and characterized with all samples having a ratio of absorbance at 260 nm and 280 nm of ≥ 1.9 but ≤ 2.1. A volume containing 0.5 μg of RNA was reverse transcribed in a 20 μL reaction using an iScript cDNA synthesis kit (Bio-Rad, Hercules, CA).

Transcript abundance was quantified using real-time quantitative PCR, SsoFast EvaGreen reagent (Bio-Rad, Hercules, CA), and the primers presented in Table 1. An equivalent quantity of cDNA from each sample was pooled and diluted to generate a standard curve. Primers were optimized and a single PCR product was verified as follows: 1 cycle at 95°C for 3 min and 45 cycles of 95°C for 5 sec and 55°C for 5 sec, and a melt curve from 65 to 95°C by 0.5°C increments for 3 sec. Water served as a no-template control and an RNA pool was included as a no reverse-transcription control for primer optimization. Controls, standards and sample transcripts were amplified as follows: 1 cycle at 95°C for 3 min and 45 cycles of 95°C for 5 sec and 55°C for 5 sec. Reaction efficiencies were between 90 and 110% based on standard curve analysis. All samples, standards, and controls were analyzed in triplicate and the quantification cycle (Cq) data were transformed using the standard curve method within Bio-Rad CFX Manager Software (version 3.1, Bio-Rad, Hercules, CA). The transcript abundance of potential reference genes was interrogated to ensure the absence of a treatment effect. Data for genes of interest were then normalized to the corresponding geometric mean transcript abundance of three reference genes: 18S ribosomal RNA (***18S***), alpha-1-microglobulin/bikunin precursor (***AMBP***), and hydroxymethylbilane synthase (***HMBS***).

**Table 1:** Primer sequence used for quantitative real-time PCR.

Gene of interest	GenBank accession	Position	Sequence (5’-3’)
*18S*	NR_036642.1	Forward	ACCCATTCGAACGTCTGCCCTATT
		Reverse	TCCTTGGATGTGGTAGCCGTTTCT
*AMBP*	NM_173989.3	Forward	ACTGTCAAGCTCTATGGGCG
		Reverse	CCTCTGTCGGGCATTGTGAA
*HMBS*	NM_001046207.1	Forward	GATGGGCAACTGTACCTGACT
		Reverse	TGGTTTGCATGGTGTCTTGC
*PC*	NM_177946.4	Forward	CCACGAGTTCTCCAACACCT
		Reverse	TTCTCCTCCAGCTCCTCGTA
*PEPCKc*	NM_174737.2	Forward	AACCTGGCCATGATGAACCCTACT
		Reverse	ACTCCTTGCCCTTCCAGGAAATGA
*PEPCKm*	NM_001205594.1	Forward	TGACTGGGCAAGGGGAGCCG
		Reverse	GGGGCCACCCCAAAGAAGCC
*G6PC*	NM_001076124.2	Forward	TGATGGACCAAGAAAGATCCAGGC
		Reverse	TATGGATTGACCTCACTGGCCCTCTT

Because calves were not fasted before hepatocyte isolation, carbohydrate status may have differed between cell preparations. In order to have normalized comparisons of treatments across cell preparations, arbitrary units of transcript abundance were expressed relative to the lowest CC, DLM, no FA treatment within each cell preparation before statistical analysis.

### Statistical analysis

Data were analyzed for treatment effects using the MIXED procedure of SAS 9.4 (SAS Institute Inc., Cary, NC) in a model that accounted for the fixed effect of treatment, all two-way and three-way interactions, and the random effect of cell preparation. Non-symmetrical linear and quadratic contrast statements were applied to test the effect of increasing concentration of CC and DLM. Given the experimental design, cells were exposed to each methyl donor treatment with and without FA, allowing for further investigation of the potential methyl donor by FA interactions. When the 2-way interaction between methyl donor and FA was *P* ≤ 0.20, the interaction was partitioned into 3 contrasts: linear without added FA, linear with FA, and difference of slope. Linear, quadratic, and the two-way interaction contrasts were considered significant when *P* ≤ 0.05 and marginally significant when 0.05 < *P* ≤ 0.10. Data are reported as least-squares mean and standard error of the mean.

## Results and discussion

A companion paper has been previously published that described an experiment conducted on the same cell preparations detailed here [21]. The primary objectives of that experiment were to examine the regulation of genes controlling methyl group transfer and lipid metabolism, and quantify VLDL and reactive oxygen species (**ROS**) secretion in response to increasing concentrations of CC and DLM in the absence and presence of FA [21]. Relevant to the current discussion, we reported that increasing CC increased the export of VLDL and decreased ROS secretion, while DLM did not affect VLDL or ROS [21]. The objectives of the current experiment were to investigate the regulation of key genes controlling gluconeogenesis, and to quantify cellular glycogen as well as glucose and BHB export in response to increasing concentrations of CC and DLM in the presence or absence of a FA challenge. The connection between favorable alterations in liver function, such as increased gluconeogenesis, protein synthesis, or ammonia detoxification, and positive post-partum production responses to peripartal supplementation of RPC and RPM is not clearly defined. In nonruminants, hepatic oxidative stress, inflammation, steatosis, and lipidosis is induced by dietary choline and Met deficiency [30]. The transition period in dairy cows is characterized by similar liver dysfunction that is credited for limiting animal performance and health and may be related to deficiencies in choline and Met when requirements are increased by the onset of lactation [31]. Improved hepatic function, particularly increased gluconeogenesis, with RPC or RPM could be a result of their hepatoprotective action that indirectly alters gluconeogenic pathways or direct actions of the nutrients on enzyme regulation or precursor availability. This would be difficult to elucidate in vivo, given animal variation and confounders that are inherent to whole-animal experiments including nutrient status, endocrine signaling, and liver function or health. Therefore, the present study was conducted in primary bovine neonatal hepatocytes because cultured cells maintain pathway responsiveness [20,32] and demonstrate species-characteristic regulation of gluconeogenesis [33–35] and gluconeogenic capacity [36].

As in the companion experiment [21], no three-way interactions between the nutrients and FA treatment or two-way interactions between CC and DLM were detected. The lack of interaction may suggest preferential use of the nutrients and independent effects of CC and DLM on gluconeogenic pathways in primary bovine neonatal hepatocytes. Because of this, observations in response to CC and DLM are discussed separately.

### Regulation of gluconeogenesis in response to Met

**Fig 1:**
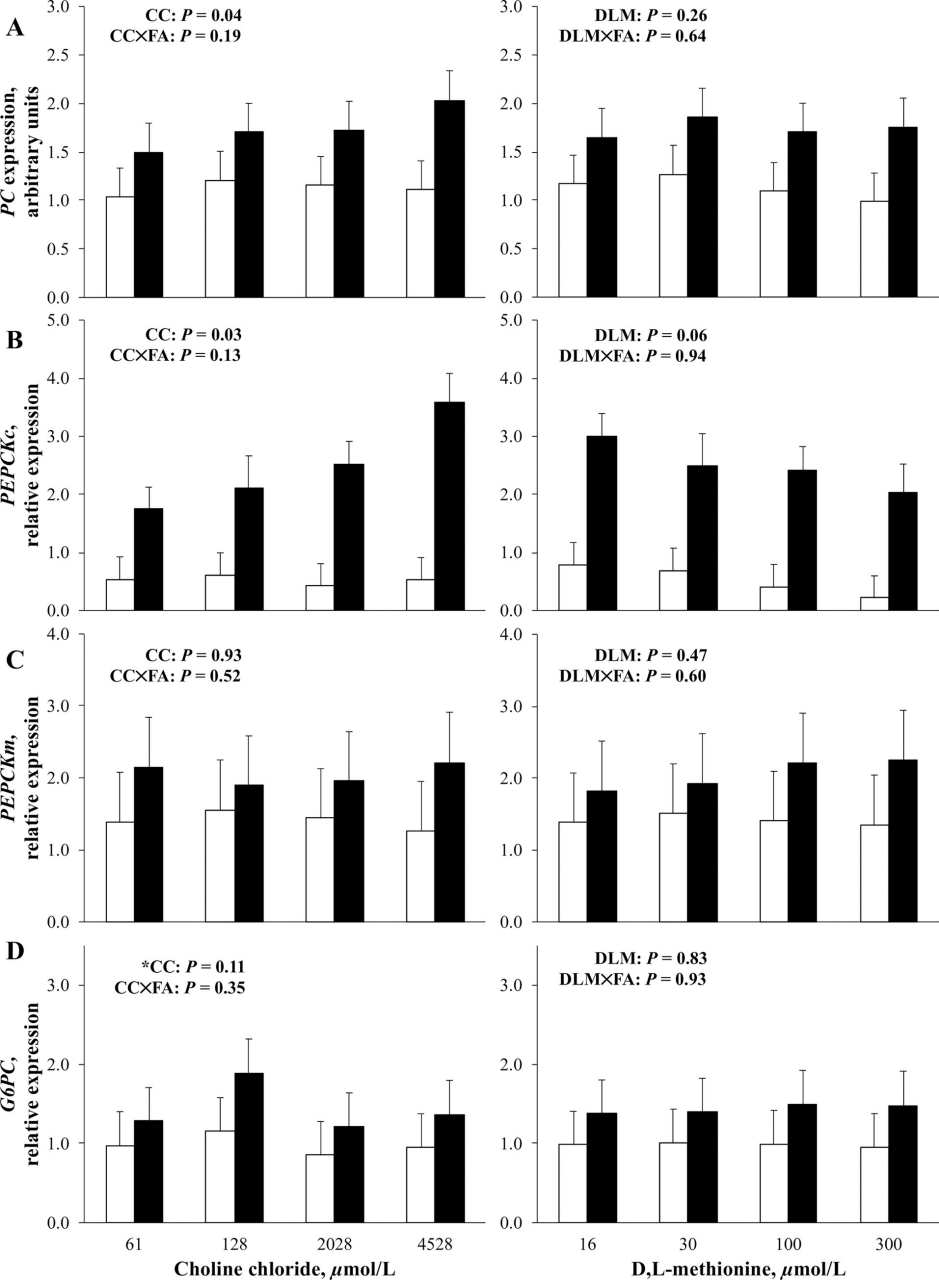
Expression of genes controlling gluconeogenesis in primary bovine neonatal hepatocytes exposed to increasing concentrations of choline chloride (CC) and D,L-Met (DLM) for 24 h, without (open bars) or with (closed bars) a 1 mmol/L fatty acid (FA) cocktail. The *P*-values for linear effects of CC and DLM are shown. Values are least squares means, with SE represented by vertical bars. *Tendency for quadratic effect of CC (*P* = 0.06). Interactions between CC and DLM were not detected: *P* = 0.65, *P* = 0.50, *P* = 0.46, and *P* = 0.81 for panel A, B, C, and D, respectively. Effect of FA for panel (A), (B), (C), and (D) was *P* < 0.01. For panel (A) and (B), when the CC and FA interaction was *P* ≤ 0.20, the potential interaction was partitioned into three contrasts: a) linear without added FA, b) linear with FA, and c) difference of slope. Results of contrasts for (A) were: a) *P* = 0.97 b) *P* = 0.005 c) *P* = 0.03 and (B) were: a) *P* = 0.89 b) *P* = 0.006 c) *P* = 0.02. Pyruvate carboxylase (*PC*), cytosolic phosphoenolpyruvate carboxykinase (*PEPCKc*), mitochondrial phosphoenolpyruvate carboxykinase (*PEPCKm*), glucose-6-phosphatase (*G6PC*).

The effect of DLM on gene expression of enzymes regulating gluconeogenesis in the absence and presence of FA is presented in Fig 1. Two-way interactions between DLM and FA were *P* > 0.20 for the expression of all genes examined, cellular glycogen, media glucose, and BHB export suggesting that the effects of DLM were similar in the absence and presence of FA. Increasing DLM did not appear to alter (*P* > 0.10) the relative expression of pyruvate carboxylase (***PC***), mitochondrial phosphoenolpyruvate carboxykinase (***PEPCKm***), or glucose-6-phosphatase (***G6PC***) (Fig 1); however, we observed a marginal linear decrease (*P* = 0.06) in *PEPCKc* expression as DLM increased (Fig 1). In agreement with the current study, a similar primary bovine hepatocyte experiment observed a decrease in *PEPCKc* expression with unchanged *PEPCKm*, *PC*, and *G6PC* as DLM was incrementally increased from 0 to 60 μmol/L [19]. Contrary to the pattern of expression observed in the previous work [19] and the current experiment, an in vivo study observed increased *PEPCKc* and decreased *PC* expression in transition cows supplemented RPM compared to controls [37]. It was proposed that in vivo results were related to increased intake of RPM-supplemented cows compared to control because increased *PEPCKc* was only observed postpartum when propionate supply would have been increased by greater intake [19]. Given that propionate has been demonstrated to regulate the *PEPCKc* promoter [35,38,39], this could have induced the expression of *PEPCKc* in RPM-supplemented cows [19].

**Fig 2:**
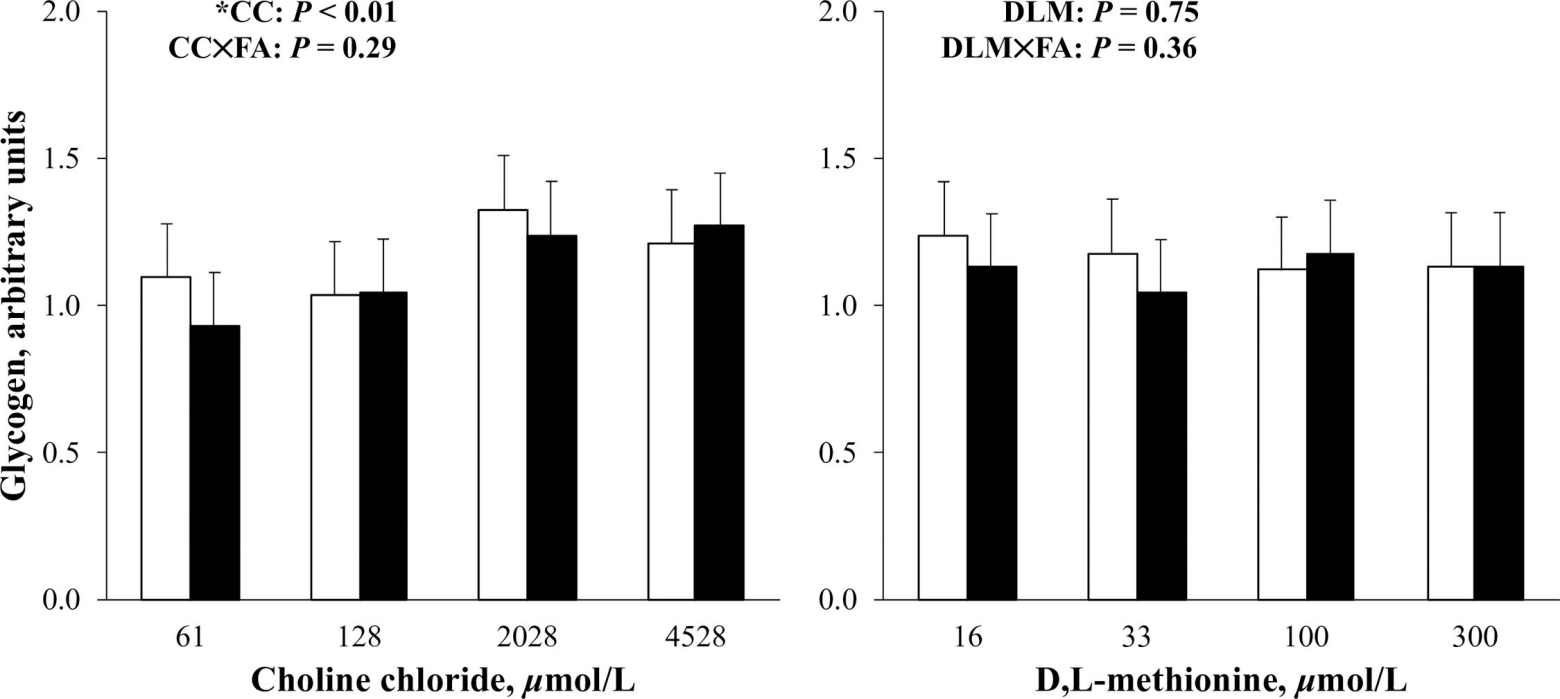
Relative cellular glycogen in primary bovine neonatal hepatocytes exposed to increasing concentrations of choline chloride (CC) and D,L-Met (DLM) for 24 h, without (open bars) or with (closed bars) a 1 mmol/L fatty acid (FA) cocktail. Glycogen was normalized to DNA and expressed relative to the lowest CC and DLM treatment without FA within cell preparation. The *P*-values for linear effects of CC and DLM are shown. Values are least squares means, with SE represented by vertical bars. Increasing CC linearly increased (*P* < 0.01) glycogen and treatment with DLM had no effect (*P* > 0.10). *Quadratic effect of CC (*P* < 0.01). No interaction between CC and DLM was detected (*P* = 0.16). No effect of FA was detected (*P* = 0.29).

**Fig 3:**
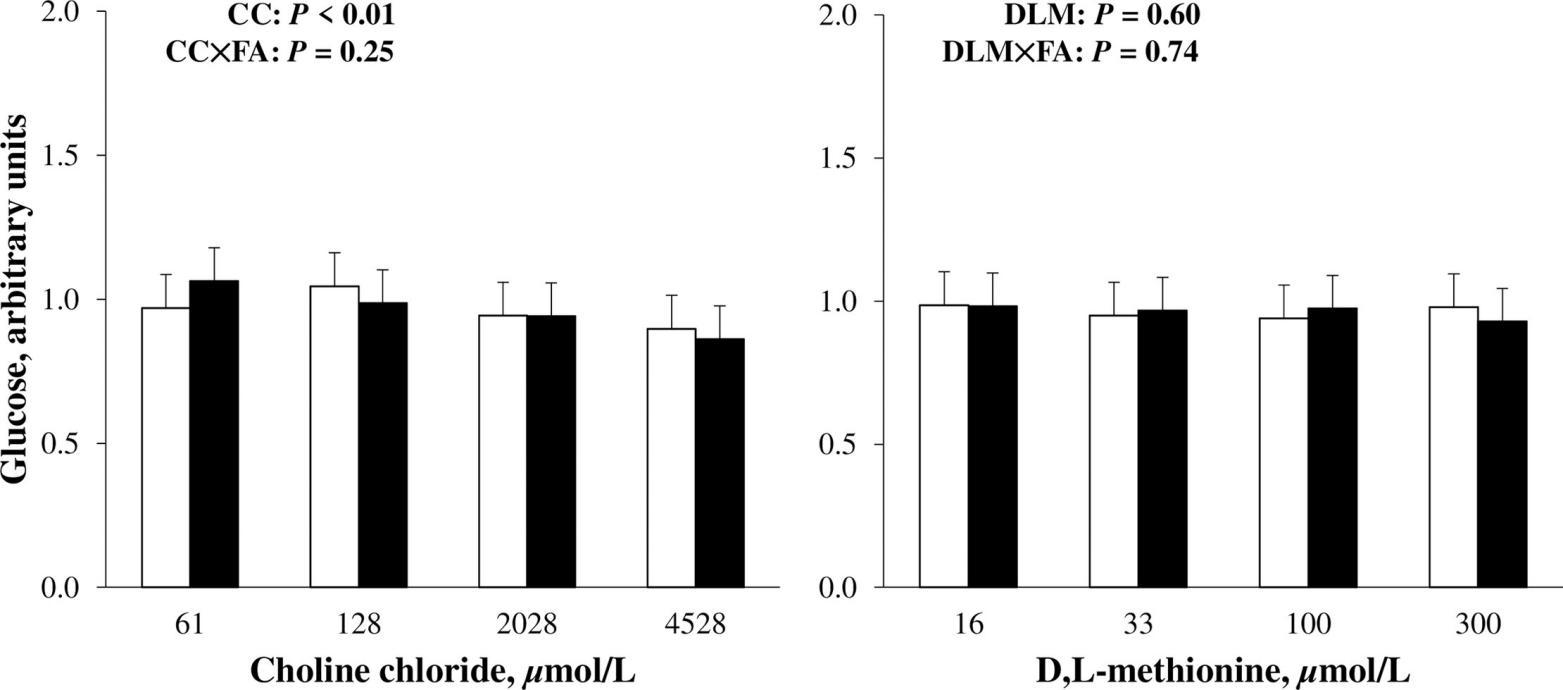
Relative glucose in cell culture media that incubated primary bovine neonatal hepatocytes exposed to increasing concentrations of choline chloride (CC) and D,L-Met (DLM) for 24 h, without (open bars) or with (closed bars) a 1 mmol/L fatty acid (FA) cocktail. Glucose was normalized to DNA and expressed relative to the lowest CC and DLM treatment without FA within cell preparation. The *P*-values for linear effects of CC and DLM are shown; no quadratic effects were detected. Values are least squares means, with SE represented by vertical bars. Increasing CC linearly decreased (*P* = 0.0001) glucose concentration in media and treatment with DLM had no effect (*P* > 0.10); there was no interaction between CC and DLM detected (*P* = 0.80). No effect of FA was detected (*P* = 0.98).

The effects of DLM on cellular glycogen and media glucose are presented in Figs 2 and 3. Increasing DLM did not appear to affect (*P* > 0.10) cellular glycogen or glucose in culture media (Figs 2 and 3). Methionine is considered a gluconeogenic amino acid as its metabolism can be traced to succinyl-CoA for conversion to OAA and then to phosphoenolpyruvate by PEPCKc or PEPCKm for use in gluconeogenesis [40]. Because the activity of PEPCKc, rather than PEPCKm, is said to regulate the entry of amino acids into gluconeogenesis [1], decreasing *PEPCKc* with increasing DLM would suggest the amino acid was not used to support gluconeogenesis. Although gluconeogenesis from Met is possible, less than 2.5% of essential amino acids contributed to glucose carbon in transition cows [41] and expression of methylmalonyl-CoA mutase, the enzyme that converts methylmalonyl-CoA to succinyl-CoA, was not affected by increased Met supply in lactating cows [42]. Biological priorities for the limiting amino acid, such as milk protein synthesis, may take precedence before increased gluconeogenesis is realized.

**Fig 4:**
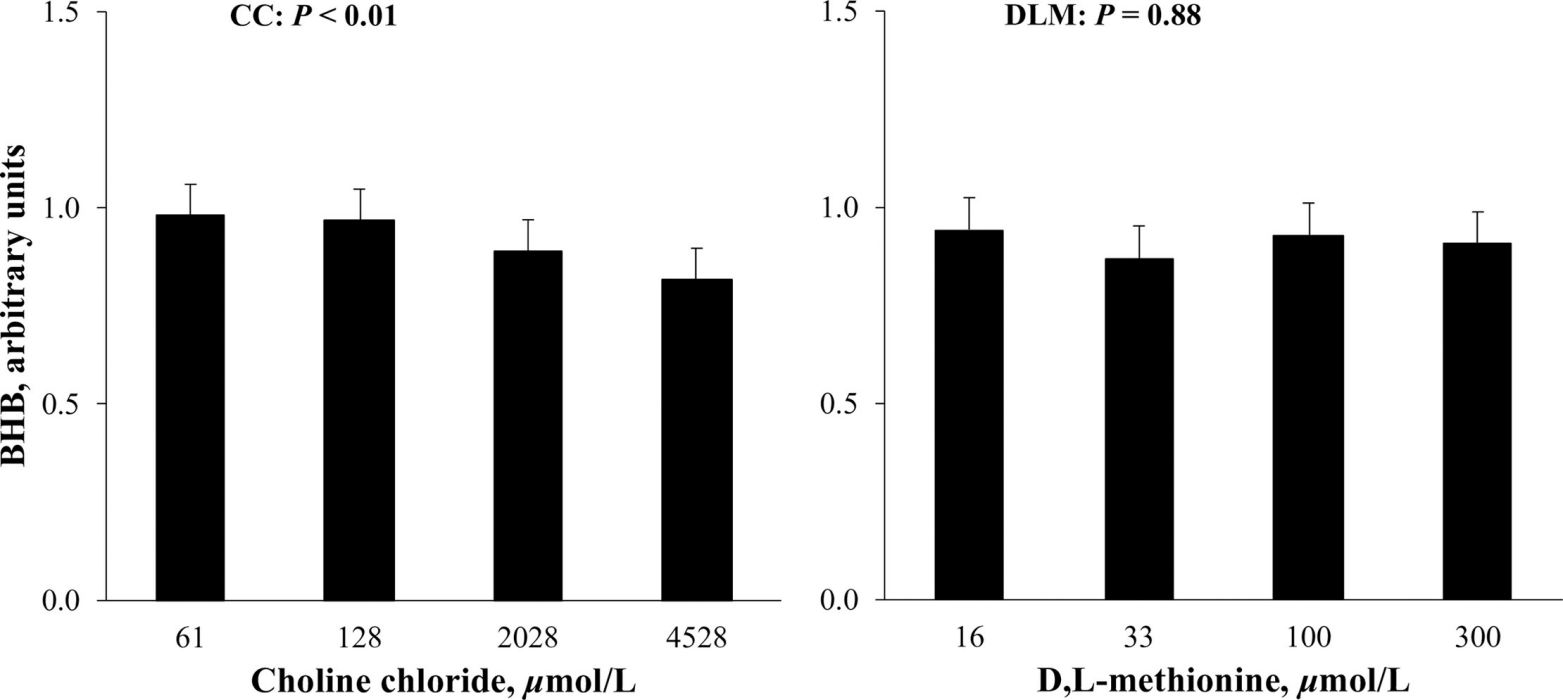
Relative β-hydroxybutyrate (BHB) export from primary bovine neonatal hepatocytes exposed to increasing concentrations of choline chloride (CC) and D,L-Met (DLM), and a 1 mmol/L fatty acid (FA) cocktail for 24 h. Export of BHB was normalized to DNA and expressed relative to the lowest CC and DLM treatment with FA within cell preparation. The *P*-values for linear effects of CC and DLM are shown. Values are least squares means, with SE represented by vertical bars. Increasing CC linearly decreased (*P* < 0.01) BHB export and treatment with DLM had no effect (*P* > 0.10). No interaction between CC and DLM was detected (*P* = 0.23).

Short-term allosteric activation of PC by acetyl-CoA is documented [43], but sustained enzyme activity changes are mirrored by changes in mRNA abundance [44–46] which is highly correlated to enzyme activity in the liver of cows [3]. Similarly, PEPCKc activity in cows was highly correlated to mRNA abundance of the *PEPCKc* gene [47]. Given strong correlation between mRNA abundance and enzyme activity of PC and PEPCKc [3,47], decreased *PEPCKc* expression while *PC* was unchanged could suggest an increase in the PC to PEPCKc ratio. This may lead to an increase in the OAA pool and therefore capacity for complete oxidation of FA in the TCA cycle [19]. Given potential increased oxidative capacity with increasing DLM, it was of interest to quantify BHB export from hepatocytes as a potential indicator of incomplete oxidation of FA. When BHB was quantified in media that incubated cells exposed to 1 mmol/L FA, BHB export did not appear to be affected (*P* > 0.10) by increasing concentration of DLM (Fig 4). Unchanged BHB export is consistent with previous experiments that did not report a difference in serum BHB when RPM was supplemented during the transition to lactation period [8,15,48]. The fact that *PEPCKc* exhibited similar results in the absence and presence of FA suggests the change was not in response to a need for oxidative capacity. The inhibitory effects of DLM on *PEPCKc* in this and a previous study [19] may have been mediated by molecular mechanisms requiring further exploration.

### Regulation of gluconeogenesis by choline

Changes in mRNA expression in the absence and presence of FA as CC was increased are presented in Fig 1. The two-way interaction between CC and FA was *P* ≤ 0.20 for the expression of *PC* and *PEPCKc* (Fig 1). When the potential interaction was partitioned into three contrasts, we observed a significant difference of slope for *PC* (*P* = 0.03) and *PEPCKc* (*P* = 0.02), indicating the effect of CC was not similar in the absence and presence of FA. Increasing CC appeared to linearly increase (*P* < 0.05) the relative expression of *PC* and *PEPCKc* only in the presence of FA (Fig 1), while mRNA expression was not affected by CC in the absence of FA. The relative expression of *PEPCKm* was not affected (*P* > 0.10) by CC, although *G6PC* expression was marginally affected (*P* = 0.06) in a quadratic manner (Fig 1).

Changes in the expression of *PC* and *PEPCKc* with increasing CC suggest the nutrient can influence gluconeogenic pathways; however, the data suggest the ability of CC to increase *PC* and *PEPCKc* interacts with FA metabolism in hepatocytes. In the companion experiment, increasing CC increased the export of VLDL in the presence of FA [21], potentially decreasing the accumulation of lipids in hepatocytes. Although cellular TG was not quantified in these experiments, decreases in liver lipids have been observed with in vivo supplementation of RPC [9,14]. Choline supply may have supported an increase in *PC* and *PEPCKc* expression by limiting the accumulation of TG in hepatocytes. Increasing post-ruminal supply of choline from 0 to 6 to 12 g/d in vivo did not appear to affect the expression of *PC* or total *PEPCK* [4]. In a separate experiment, RPC stunted the characteristic postpartum increase in *PC* expression and did not affect *PEPCKc* in cows supplied 14 g/d of choline post-ruminally [17]. Similar to RPM supplementation, the effects of RPC supplementation on expression of genes in vivo may interact with intake and energy balance during the peripartum period.

It is important to note that G6PC is regulated both transcriptionally [49,50] and post-translationally [51] in nonruminants. For these reasons, glucose and glycogen were quantified directly in order to determine potential change in the output of gluconeogenesis by treatment. The effect of CC on cellular glycogen and glucose in media is presented in Figs 2 and 3. Increasing CC linearly and quadratically increased (*P* < 0.01) cellular glycogen (Fig 2), but decreased glucose (*P* < 0.01) in media (Fig 3). The effect of CC on cellular glycogen did not differ (*P* > 0.10) by FA treatment (Fig 2). Glycogen, a storage form of glucose in liver and muscle, is critical to the supply of glucose to prevent hypoglycemia during fasting [52]. In transition cows, liver glycogen stores are quickly depleted as calving approaches, reaching a nadir immediately [3] or shortly after calving [53], and takes several weeks to return to prepartum content. Increased liver glycogen with increasing supplementation of RPC have been observed in early lactation cows experiencing fatty liver [54] and non-lactating cows feed-restricted to induce hepatic lipidosis [14]. During the transition period, liver glycogen has been negatively correlated with severity of hepatic lipidosis [55] and increasing the ratio of liver TG to glycogen is suspected to increase ketosis susceptibility [56]. Liver glycogen is similarly depleted in rodent models of fatty liver induced by Met and choline-deficient diets [57]. Supplemental dietary betaine, a metabolite of choline, reduced liver lipids and increased liver glycogen in a mouse model of non-alcoholic fatty liver disease [58]. Because previous work suggested that fatty liver inhibits gluconeogenesis in bovine hepatocytes [59], it was reasoned that lower liver TG in cows fed increasing amounts of RPC may have allowed for greater rates of gluconeogenesis [54]. This potentially spared glycogen from hydrolysis for use as a glucose source or replenishing glycogen more quickly than cows not receiving RPC [54].

The ability of RPC in vivo and CC in vitro to increase hepatocyte glycogen may be the result of decreased lipid infiltration; however, the combined effects of RPC to increase liver glycogen during ad libitum feeding in the absence of lipid infiltration [14] and CC to increase cellular glycogen in hepatocytes not exposed to FA in the present experiment suggest choline more directly affects cellular glycogen. In nonruminants, glycogen is primarily synthesized from blood glucose [52] and is partially regulated by the activity of glucokinase, the enzyme that phosphorylates glucose to sequester it intracellularly [60]. As bovine liver appears to lack glucokinase activity and net uptake of glucose [61,62] and exhibits negligible rates of extracellular glucose catabolism [63], changes in cellular glycogen in bovine hepatocytes in the present experiment likely reflects storage of newly synthesized glucose-6-phosphate (**G6P**) via glyconeogenesis [52]. It is possible that accumulation of G6P as glycogen, instead of release as free glucose, may reflect the in vitro conditions that do not fully mimic glucose demands of peripheral tissues.

If glycogen carbon came from gluconeogenesis, it is also possible that CC served to repartition glucose precursors. The use of methyl groups derived from choline, after oxidation to betaine and conversion to dimethylglycine, is directly tied to the metabolism of glycine and serine, two glucogenic amino acids [64] that are also involved in supplying the folate methyl pool [18]. In the companion experiment, gene expression of the transmethylation pathway suggested that CC stimulated the regeneration of Met with methyl groups derived from the folate pool [21]. If CC increased carbon transfer in the folate pool and supplied methyl groups, increased rates of glycine synthesis from dimethylglycine or decreased rates of serine catabolism for methyl groups could have increased their availability for use as glucose carbon or stimulated glycogen synthesis. Serine and choline injection [65] and glycine feeding [66] increased liver glycogen in rats and should be further examined within this context.

The potential for CC to decrease hepatocyte TG by supporting VLDL export may have affected FA oxidation. In contrast to DLM, increasing the supply of CC decreased (*P* < 0.01) export of BHB from hepatocytes (Fig 4). In vivo, RPC has either decreased [48,67] or did not appear to affect circulating BHB in postpartum dairy cows [9,54,68]. Choline may have decreased the export of BHB by decreasing the supply of FA for oxidation as more FA were exported as the TG component of VLDL.

### Fatty acid treatment upregulated gene expression

Because cell culture models should attempt to mimic in vivo physiological conditions, a 1 mmol/L FA cocktail was exposed to cells that mimicked the profile and concentration of FA observed in circulation at the time of calving [25]. This was done to more closely simulate a transition cow scenario and the hepatocyte dysfunction induced by lipidosis that may be mitigated by choline and Met, and therefore, interact with their ability to affect glucose metabolism. The effect of FA treatment on expression of genes of interest is presented in Fig 1. In the present experiment, FA treatment increased (*P* < 0.0001) the relative expression of all genes investigated. The effect of FA on genes controlling gluconeogenesis was not likely a result of global transcriptome upregulation, as FA treatment differentially altered the expression of genes controlling methyl carbon and lipid metabolism in the companion experiment [21]. Upregulation of gluconeogenic genes examined here could suggest a common positive transcription regulator conserved among the genes that was induced by FA treatment; however, it is possible that individual FA or separate transcription factors acted independently to upregulate each gene. Gluconeogenic stimulation by FA has been well characterized in nonruminants [69,70]; however, the upregulation of all gluconeogenic genes examined in the current experiment by FA treatment was unexpected. Previous incubation of Madin-Darby bovine kidney (**MDBK**) cells with cocktails of FA observed prepartum, at calving, or at calving in cows with induced fatty liver, indicated differential regulation of the genes that was reflective of the FA cocktail and concentration [25]. A similar 1 mmol/L FA cocktail to the one used in the present study increased the expression of *PC* and *PEPCKm*, while *PEPCKc* and *G6PC* were unchanged, in MDBK cells when compared to a 0 FA control [25]. Differences may persist between the present experiment and the previous because of differences in regulation or responsiveness of gluconeogenic enzymes between bovine hepatocytes and bovine kidney cells, despite both possessing gluconeogenic capacity.

Although the current experiment only reflects a comparison of expression in the absence or presence of FA, results suggest the expression of gluconeogenic mRNA can be induced by FA in bovine hepatocytes. Some, but not all, gluconeogenic enzymes are responsive to FA in vivo. Pyruvate carboxylase is increased at the onset of lactation [3,71] and during feed restriction [72], mediated by FA regulation of the gene promoter [73,74]. Expression of *PC* is also higher in cows that have greater circulating FA peripartum [71,75]. The expression of *PEPCKc* is hormonally and energetically regulated [76] and appears to be less variable at calving or during feed restriction in dairy cows when circulating FA increased [3,72]. Specifically, *PEPCKc* was unchanged [3,4,17] or decreased [71] immediately after calving and expression only increased 2 to 3 wk after calving [3,4,17,71]. Time-dependent changes in *PEPCKc* expression may be related to its regulation by propionate [35,38,39]. Expression of *PEPCKm* appears less responsive to changes in physiological state, such as the transition to lactation [3,47,77], although others reported an increase in *PEPCKm* expression at calving [71]. The enzyme may be constitutively expressed in vivo [78] but was responsive to FA in the current experiment and previously in MDBK cells [25]. Expression of *G6PC* has been variable during the transition period [71,75], and may be related to in vivo regulation by glucocorticoids and insulin [25]. Differences between in vitro hepatocyte response to FA exposure observed here and in vivo may be apparent because in vitro experiments remove extra-hepatic factors that might contribute to regulation. Although FA upregulation of gluconeogenic genes could suggest an increase in gluconeogenesis, FA treatment did not affect (*P* > 0.10) glucose storage as cellular glycogen or glucose in media (Figs 2 and 3).

It is noteworthy that addition of FA to cell culture media and subsequent lipidosis did not inhibit glucose accumulation as cellular glycogen or in media. This result was unexpected as liver TG accumulation has been associated with adverse effects on liver metabolism in ruminants [79,80]. Hepatic lipidosis has been credited for limiting gluconeogenesis during the transition period and contributing to the development of hyperketonemia peripartum [5,81]. Lipid infiltration induced by prepartum overfeeding decreased the enzyme activity of total PEPCK in postpartum cows [82]. Liver slices from cows feed-restricted to induce hyperketonemia [79] and primary hepatocytes from ruminating goats [80] that accumulated TG in vivo exhibited decreased gluconeogenic capacity from propionate, lactate, and alanine. Hepatic lipidosis and hyperketonemia are associated with hypoglycemia in vivo [5]; however, causality has not been clearly defined and decreased gluconeogenesis ex vivo from liver slices described above may have been confounded by the conditions that led to lipidosis and or hyperketonemia in vivo [79,80]. Triglyceride accumulation may not directly limit gluconeogenesis; however, the capacity to detoxify ammonia is reduced by lipidosis [83] which appears to limit gluconeogenesis from propionate [84]. In the present study, FA exposure and lipid accumulation did not appear to inhibit gluconeogenesis when pyruvate was included as the primary gluconeogenic precursor. Given that the present study was not designed to quantify rates of gluconeogenesis, molecular strategies that employ labeled substrates and isotope tracing may more accurately evaluate the effects of FA, as well as choline and Met, on flux through pathways of glucose and energy metabolism in primary bovine hepatocytes.

## Conclusions

In agreement with our hypothesis, CC and DLM differentially affected key genes regulating gluconeogenic pathways in primary bovine hepatocytes. The inhibitory effect of DLM on *PEPCKc* expression without decreasing glycogen or glucose in media should continue to be investigated. Combined increases in *PC* and *PEPCKc* with added CC support an increase in gluconeogenesis and storage of cellular glycogen in the presence of FA. The potential mechanism of CC to increase cellular glycogen when mRNA expression was unaltered in the absence of FA warrants further study.

## Supporting information

S1 Table Data table. (XLSX)
